# Editorial and Miscellaneous

**Published:** 1862-10

**Authors:** 


### EDITORIAL AND MISCELLANEOUS.

*Volunteer Surgeons.*—It would appear that our brethren in the army have no rights which their official superiors are bound to respect. Among other instances that of Surgeon David S. Hayes, Penn. 16th Vol., seems to have been one of peculiar hardship. The secular press have taken up the matter as an example of censurable indifference to trust, and coincidently indulged in many coarse expletives applied to the medical staff generally. We deem it no more than justice that his own explanation be given to the public. It will be recollected that the, formerly very much be-puffed, Secretary of War, some time since summarily removed, without any permission of explanation, Surgeon Hayes, for leaving a car load of wounded men on their arrival at Washington, late at night, and going to a hotel for supper and rest. Thus much the Secretary listened to and no more, ordering the papers of dismissal at once issued. A correspondent of the Philadelphia *Inquirer* writes as follows:

"The friends of Dr. Hayes think that he has been most hastily judged. They say that he telegraphed the War Department that the train would reach here at 9 o'clock Saturday night; that the dispatch, from some cause, never reached its destination; that he arrived in the middle of a storm, at midnight, and found that no provision had been made for the sick and wounded; that he endeavored to find the proper officer, but on account of the lateness of the hour, was unable to do so; that he left the wounded in charge of the surgeons who accompanied him; that he was completely exhausted from the duties which had devolved upon him for the previous three days, and could not help taking some repose before he started out on Sunday morning to seek the Surgeon-General. There is much sympathy manifested for him, and he claims to be able fully to vindicate himself from censure."

We do not think the case needs any comment. A harder worked, poorer paid, and more abused class of men than the

Army Surgeons can hardly be found. Generally with tenfold the brains of the Colonels and Brigadiers over them, with better educations, and more gentlemanly habits, they are treated with little more respect than the orderlies. They are expected to remedy, *instanter*, by potent combinations of drugs, the result of the vices of the officers and the reckless habits of the soldiery. It is rare that commanders will pay the slightest heed to the sanitary arrangements so earnestly besought by the surgeons. They expect their own indolence and heartlessness, not to say brutality, will be neutralized by the contents of the regimental medicine chest, and if they are not, then the surgeon is cursed and calumniated.

Worn down by constant toil, days and nights of exposure and unrest, more surgeons, proportionately, have been killed and disabled by disease than of any other class in the army, and yet they are rarely or never mentioned in official bulletins, and are never promoted except to additional hardships, with no increase of emoluments, present or prospective. And honorable Secretaries insolently refuse explanations or excuses when the physical part is positively incapable of sustaining, without some little repose, the immense burden imposed upon it. Nothing but the purest philanthropy and most self-sacrificing patriotism would permit a surgeon, capable of performing the duties of the office, to accept it under the present position of affairs. We trust, by and by, to hear of a reform in this whole matter. The haggard countenance and emaciated bodies of our professional brethren who have resigned, or received brief and niggardly furloughs, with their unanimous testimony, have cured us of the last lingering remains of zeal for professional connection with the army. We are willing to shoulder musket and knapsack whenever government calls ; but the pill bags and catling not just yet.

---

*Chloroform Removing Bitterness.*—It is stated that Chloroform has the property of modifying the taste of certain bitters, and when added in water, it nearly altogether removes the bitterness. In cases where the Chloroform is not contraindicated, this little matter is worth recollection and trial.

---

*Treatment of Fevers.*—Prof. JAMES BRYAN, Brigade Surgeon, writes to the Boston *Med. Journal*, from which we extract the following:

There has been no one treatment adopted generally for typhus and typhoid fevers in this division. The expectant and stimulant have been the most common. The surgeons have relied chiefly upon what the ancients call the *naturales*; the use of whiskey punch, carbonate of ammonia, or turpentine—very little, however, of the two latter articles. Eberle's strong recommendations of the carbonate of ammonia seem to have been forgotten, and "Wood on turpentine" has been left to the mercy of caricaturing students in the halls of the "oldest medical institution" in the United States. The profession in the army, here, seem to be entirely at sea, no two of them appearing to agree on any plan of treatment. They appear to be like the Parisian philosophers, who anxiously inquired of Franklin what religion he professed, and found, much to their satisfaction, that he professed none. I have felt much dissatisfaction with this condition of practice, and have requested one of my colleagues to try, in some cases of pulmonary and cerebral congestion, *feelingly*, the now exploded lancet. His reports, thus far, have been favorable. In some cases of stupor and cerebral congestion, I have suggested the resort to one or two large doses of calomel, followed by Dover's powders and whiskey punch. These cases have done well also; and lastly, at my request, the calomel and quinine practice, ten and ten, has been resorted to where bilious remittent symptoms predominated at the beginning. This treatment seems still to hold its own in this district among the resident practitioners.

I remember well when Dr. Parry, from the valley of the Mississippi, many years ago, then a student in the Jefferson Medical College, threw a bomb shell into the profession in Philadelphia, by reading his thesis before the Medical Society, and quietly asking "what shall I do with the pulseless, moribund cases of congestive fever of the Mississippi?" Professor Meigs, in his usual enthusiastic and fanciful style, recommended "the lancet—the lancet—the lancet"! It, he said, would relieve the overloaded internal organs; it would take the weight from off the nervous centres; it would allow the

*endangium* to produce healthy blood. In a word, if the patient died, like that of the pupil of Dr. Sangrado, it would be for want of bleeding! Dr. Wood was astonished and dismayed at the doctrine, and, assisted by the brilliant but eccentric Jackson, both of the University, castigated the professor of the Jefferson Medical College most unmercifully. Prof. Wood did not approve of bleeding at all, except in rare cases, and "would treat the case according to circumstances." Professor Jackson gave a brilliant dissertation, which lasted part of two sessions of the Society, on the then newly published cell theory of Schwan and Schleiden, while Dr. Parry stated the western practice to be, in some cases, large doses of calomel, in others large doses of quinine, while others combined the quinine and calomel practice in one. Professor J. K. Mitchell took hold of this practice at once, enforcing it with his usual eloquence and point, and illustrating it in public and private practice during the rest of his life.

According to my observation, the same diseases exist on the southern coast of North Carolina, as those mentioned by Dr. Parry. Now what shall be our treatment? Will the profession of New England tell us what we shall do? Shall we use turpentine with Wood, calomel and quinine with Mitchell, or the lancet with Meigs and Rush? The hardships of the winter campaign at Hatteras, Roanoke and Newbern, have battered many of our men to a condition at least as low as that of the natives and negroes. Shall we fear this real or apparent prostration, and shun the lancet—the almost universal practice over our country for some time past? Shall we forget Hamilton's eulogies of cathartics? Shall we attempt to destroy the malarious poison in the blood by immense doses of quinine? Shall we stimulate with carbonate of ammonia, or piddle along with the turpentine emulsion? or shall we, in the language of one of our surgeons, "sock into our patients large doses of whiskey punch?" Or what shall we do?

In surgical practice, we are taught "*anceps remedium melius quam nullum.*" Is the lancet that doubtful remedy? Or is it right to see our patients die from the very beginning of the disease, without attempting to do something for their relief?

Dr. Bryan has expressed exactly the thoughts which arise in the minds of all young practitioners when called to face the reality of practice, instead of the *un*realities of ancient theorists, brilliantly imaginative lecturers and book writers.

We have not a particle of doubt that although he asks a variety of questions, he is dubious not in the least. With the experience and light of the present day, there is not room for question upon the points raised.

Bloodletting in such cases is simply murderous; calomel, in the manner suggested by Dr. B., incidentally useful, but the remedies are quinine, alcohol and nutriment. One or two full doses of calomel at the outset are almost invariably useful—the quinine and brandy *always*. Concentrated nutriment should speedily follow, or else typhoid prostration *will*. Opium is especially useful from its supporting power, its own great antiperiodic energy, and its remarkable efficiency in obviating the occasional unpleasant effects of the quinine. The indications for the use or non-use of the mercurial ought to be clear to any one competent for the position of army surgeon. Salivation in such cases is both unwarrantable and inexcusable.

---

*Bloodletting in Puerperal Convulsions.*—In the report of a meeting of the Conn. River Valley Assoc., in the Boston *Med. Jour.*, we find the following paragraph:

"Dr. Sawyer had seen several cases which were preceded by cephalalgia, in which he had recourse to copious bleeding, which resulted favorably. Dr. Crane considered the pathology to be pressure on the motor nerves, and thought this might be removed by venesection and cupping. Dr. Crowley had seen five cases, all of which had occurred after delivery. He had relied on phlebotomy and cathartics, with cold applications to the head. Dr. Gregg, of Newport, remarked that in an extensive practice of over fifty years, he had seen a good many cases both before and subsequently to delivery. He thought that in all cases where the contents of the uterus had not been removed, the first thing to be done would be to remove it. He then gave the history of several cases which illustrated this. Chloroform and ether he had never used; but for the benefit of the younger members of the profession, he would say that he had bled a patient to the extent of thirteen pounds in a day with great benefit."

To which may be rejoined that Dr. G. has practiced very wisely in removing the contents of the uterus as soon as possible; very unwisely in never having used chloroform or ether, and very "heroically" in bleeding to the extent of *thirteen pounds in a day*, the addition of the words "with benefit" being rather dubious in propriety. Prompt removal of the contents of the uterus probably was coincident with that bleeding, and, thanks to the patient's vital power, she escaped both dangers. One of the physicians present commended the mixture of chloroform with ether as having a better effect than ether alone; probably the chloroform without the ether would have been better still. It was remarked that where the effect of the anæsthetic was most powerful, the contractions of the uterus were more forcible.

The era of bloodletting as principal agent, or even frequent subordinate, in puerperal convulsions, has gone by.

---

*Sciatica.*—In a conversation before the London Medical Society (*Lancet*, July, 1862,) on the subject of sciatica, Mr. Coulson remarked that in the chronic form he found benefit from relaxing the nerve by position and contemporaneous exhibition of Iodide of Potassium. Dr. Habershon called attention to the occasional dependence of sciatica upon inflamed hæmorrhoids, which had also been noticed by Sir B. Brodie and Mr. Ashton. He confirmed the value of Iod. Potassii, and occasionally of the Bichloride of Mercury. Others adduced cases depending on Hæmorrhoids. Dr. Leared had seen cases of a strictly inflammatory sort which required the usual remedies for inflammation. He lauded arsenic as the remedy *par excellence* for sciatica and neuralgia. Mr. Hulke praised the hypodermic use of morphine. The President, Dr. Sibson, had found the sesquioxide of iron of remarkable advantage in hospital cases, commencing with a dose of turpentine and castor oil, and a blister dressed with belladonna ointment. Speaking empirically, in those cases where neither syphilis, rheumatism, nor gout was present, this plan of treatment would be found most useful.

*The Liver et alii.*—Prof. Geo. Hasley read a paper on this subject before the Royal Med. and Chir. Society, which is noticed in the *London Lancet.* He recognizes the usual distinction between jaundice from suppression, and jaundice from reabsorption of the secreted but retained bile; an abstract of the paper we published in the August number, (p. 491,) which is worthy attention. In the discussion which ensued, Dr. Marcet said the examination of the fæces would often clear up difficulties in the diagnosis of the cause of jaundice. If the bile is retained, there is in the fæces an abnormal quantity of fat, not recognizable by the eye, but extractible by alcohol. The fæces are white, and the fatty matter consists of fatty acids. This of itself showed also that the pancreas was not alone in providing for the digestion of fat—the secreted bile being necessary for the perfection of the process.

The relation of the liver to the fat elaborating process in the system, has been too largely neglected, although that is perhaps the most important part of its function. It is probable that the blood corpuscles cannot be developed without the presence of fat supplied from the liver directly, or modified by its secretion. Hence the anæmia which speedily follows suspension or impairment of its function. Hence the benefit arising from the exhibition of cod-liver and other oils, which require little digestive change before being received into the lacteals. Hence the folly of vegetarianism, which entails excessive labor upon the liver in the manufacture of fat from elemental or chemically similar substances. Hence the absurdity of the direction to carefully skim off the floating globules of fat from the surface of "beef-tea," often when the patient requires the fat more than the nitrogenized compound. Hence the necessity often for alcoholic stimulants which appropriate nutriment would have made unnecessary. Hence the necessity, when the blood-making processes are too extreme, and the formative results of exudation are too active, as in the acute stage of inflammation, of suspending or materially lessening the supply of fatty food, and even causing elimination of much of that prepared by the liver through the influence of mercurial and other cathartics.

It is common to speak of "portal venous congestion," or "hepatic venous congestion," as respectively the cause of jaundice, and the distinction is a good and valid one in one sense; but it should be recollected that congestion is rarely a mere mechanical affair. The heart, arteries, capillaries and veins constitute something more than a mere hydraulic system of tubes. Stasis of blood occurs in capillaries, arteries and veins from other than mechanical causes. The physiology of the circulation readily explains the pathology, and thus in every case of jaundice we have got to go back to the causes of the diminished or annihilated local action upon which the retention or obstruction depends. And then, seek to relieve, not solely the effect, but the cause—which will issue generally in something more than exhibition of mercurials, alkalies, cathartics, podophylline or inspissated bile.

To one who has carefully kept pace with the discoveries of Physiology, there are few subjects which are of greater interest than the relations of fat to the blood and the general system.

The well known milky appearance of the chyle in the lacteals is clearly due to the particles of oil which every where in solutions of albumen condense to themselves a vesicular coating from that substance. Here is the first step in that wonderful cytogenesis which eventually develops the blood or "liquid flesh." The lacteal glands aid in the process by bringing the chyle in intimate contact with developed blood. The direct effect is unknown in its essence, and it is needless to speculate upon it. The undeniable fact remains, that without the aid of oil or fat there is no cell.

The albumenoid vesicle containing fat, is impregnated or vivified by the dotted particles derived from the gland. Some escape, and dissolving yield up their contents to furnish nutrition to their germinant fellows.

The alkaline constituents of the blood (chyle, or lymph) are the means of this solution.

It would appear that fat is also necessary to the genesis of fibrin, as that depends upon the perfection of the blood corpuscle.

The stomach of Alexis St. Martin is chargeable with begetting great error in physiological notions. Digestion is perfect, or the reverse, according as it occurs in its proper place. Nitrogenized food is freely dissolved and absorbed from the gastric cavity,—oils and fats but to a very limited extent. It is probable that the small amount here absorbed is received by the minute villi as discovered by Dr. Neill (Vid. Am. Jour. Med. Sci., v. xxi, p. 13.) In like manner the duodenal and other intestinal mucus membrane by its blood vessels absorbs such portion of the albuminous food as may have escaped gastric absorption. Thus fats and oils are easily digestible in their proper place, which is *below* the stomach even when as readily examined as that of Alexis St. Martin.

Both observation and experiment show that there is within the system a chemistry which will convert not only saccharine, but even nitrogenized food into fat when little or none of that substance is taken as food. This phenomenon is entirely analogous to the production of wax by bees fed almost exclusively upon sugar. They will produce three times as much wax as the amount previously contained in their bodies or in the food taken. But some little wax must be taken in, or they will not manufacture any.

It is quite clear that the liver is the fat laboratory in the animal man. The fatty matter of vegetables in itself is insufficient to supply the requirements of histogenesis and repetition, but its presence in the portal blood favors the conversion of compounds which contain its elements, so that the bile and blood of the hepatic veins contain a very considerable amount. When this conversion is not required as in the case of animals freely supplied with oily food, there is much less bile secreted. Lehman discovered that in animals undergoing starvation there is an excess of fat and a deficiency of sugar in the portal vein, but that the condition is inversely changed in the hepatic vein.

The degradation of tissue is usually, if not invariably, accompanied with fat deposit instead of previous nitrogenized substance. Hence in the bodies of those who have died of inanition it is always noticed that the gall bladder is full of bile*—the blood bringing to the liver the result of fatty degeneration of the tissues, this being secreted partly with the bile, and partly, perhaps, converted into cholic acid and sugar.

The demand for sugar is for sustaining the animal heat; the demand for fat is primarily for the evolution of blood-cells, and secondarily for decomposition into cholic acid and sugar. The fatty liver furnishes little bile, but much sugar—the separation of the fat from the blood favoring the evolution of sugar, but the hepatic tissue having insufficient cell energy to further eliminate bile.

It need scarcely be remarked that fatty degeneration is produced—first, by a wrong or defective state or composition of the blood; second, by an insufficient supply of blood; third, by a deranged or obstructed influence of the nervous system, (eventuating, of course, and being properly included in the first cause), and fourth, by an imperfect, unhealthy or declining state of the part to be nourished.

There is hence no difficulty in seeing that though the cells of the liver may be gorged with oil, yet an insufficient quantity finds its way to the lacteals and the blood. Meantime sugar (liver-sugar) is produced in disproportionate amount, and being oxidated in all the tissues and cells develops more or less febrile heat.

In the healthy condition of the various organs when supplied with their appropriate food, the production of the various secretions we know to be uniform; but man is exposed to almost every variety of condition and food. The proportions must be maintained among the various constituents of the blood, or disease will ensue.

* Does not this explain why mechanical distension of the stomach, &c., with innutritious substances relieves the sensation of hunger—the bile being thus poured out into the duodenum and its fatty portion absorbed?

From our present knowledge upon this subject we may look upon the liver as a great regulator in the mechanism of nutrition.

It provides oil from substances which contain but its elements,—hence man can live without taking it directly as food, but as other organs which are called to excessive labor, so this in time must become diseased from this cause alone.

It unquestionably derives some adeps from the disintegrating blood cells brought by the splenic vein, the hæmatin of the old cells by deoxidation passing to the form of the coloring constituent of the bile,—the iron and oxygen in great part passing to the nutrition of the younger corpuscles. It may elaborate liver-sugar for respiratory uses, and, finally, eliminates an unstable chemical portion whose retention in the blood would be productive of serious mischief.

It has become quite fashionable of late to decry all allusions to diseases of the liver as decidedly old-fogyish or behind the times.

Dexterity in percussion, and oracular shaking of the head after applying the stethoscope bauble, are deemed sufficient substitutes for acuteness of penetration in discovering the causes of disease.

The urine, too, has its worshippers, and the test tube stand, spirit-lamp and meter (all well enough in their way) are considered worthy of supplanting the poor old liver of the fathers.

Nevertheless the liver is and must remain one (if not *the*) of the most important objects of diagnostic and prophylactic study. It is the prominent intermediate agent in converting the inert and lifeless to the active and vitalized.

Basing therapeutics upon the views herein set forth, it is seen that the range of administration of the fats and oils becomes exceedingly widened. They are not directly calorifacient, but they are necessary to the building up of heat carriers. They can thus be administered in a vast number of cases *where there is heat of the surface, but poverty of the blood.*

Thus in hectic and typhoid fevers and in the latter stages of all phlegmasiæ, when, as the older writers would say, there is heightened irritability with lessened tone.

Vegetable decoctions, wherewithal these diseases are continually plagued in common treatment, yield sugar and consequent heat or vitiated secretions,—the fats and oils bring living cells into action whereby the regular transmutations and developement of each and every tissue arise.

In tuberculosis this is peculiarly the case. Tuberculosis is the manifestation of diseased glandular action, in every case intimately connected with the non-reception or development of fat.

In the child the mesenteric glands and intestinal villi fail to develop the corpuscle or receive the oil.

In later life more frequently vitiated appetite and faulty teaching or habit disqualify the liver for performing its function.

The vegetarian*ism*, and the "low diet" dogma, implant phthisis in the systems of thousands, and tamely call it "hereditary" as though *vices* are necessarily so.

Consider the vast amount of oil which is secreted upon the investing integument and the waste to which it is subjected by hydropathic scrubbing.

Look at the delicate skin of the consumptive and the rapid oxygenation which is momentarily sapping the foundations of life. Then the disordered appetite, which *secundem artem* from a "dear dyspepsia grows a dire disease."

Inunction of oleaginous substances will, in the cases previously adverted to, afford vastly more relief than the water douching and sponging so much practised. *En passant*,—this is a remedy of much greater efficiency than commonly supposed. In all cases of poverty of blood corpuscles, but more especially in tuberculous cachexia, it is of wonderful efficacy.

In scarlatina its power is well acknowledged, but its *rationale* has not hitherto been satisfactorily explained.* No other

* A part of this article was published by the writer in June, 1857. This remark was more especially applicable at that time than now. But the whole subject has been practically too much ignored.

remedy will like it prevent the rapid decomposition of blood corpuscles, so commonly followed in this disease by dropsy or renal disturbance. The fat favors the development of energetic blood cells from albumen which otherwise is strained off in dropsical or urinary flux.

During its operations the lymphatics become true lacteals, and the lymph will grow milky like chyle, though not to the same extent.

Contrary as it may apper to received opinions, the emaciation and flux of diabetes are alleviated by it. Internally administered, there is theoretic danger of conversion by diseased hepatic action into sugar and cholic acid, but practically my own experience is favorable to its free use in connection with nitrogenized animal food.

The catalogue of applications, both externally and internally, might be extended to the verge of nosology, but is sufficient that we recognize the principle involved.

There is no so-called *tonic* medicine which can exert more than the merest modicum of the impression, capable of being produced by this active class of alimentary articles.

The antiphlogistic regimen essentially consists in counteracting the effects of the received adeps. Abstract oil and you simultaneously take away red corpuscles or oxygen carriers and fibrin producers. Bloodletting does the same. Cholagogues, as mercury, etc., urge the bile too rapidly along the intestinal tract to allow absorption of its hydrocarbon and resulting increased activity of cytogenesis.

Moderately administered they promote it by augmenting the conversion by the liver of this genetic medium and agent. When carried to excess, there is resulting cachexia from poverty of the blood in cells, and too abundant formation of liver sugar and hence atonic fever.

In warm climates the production of liver fat is nearly or quite sufficient for the purposes of blood genesis. Here there is danger of excess, as is shown by the ready and profuse flux of bile. As with the formative cells of the vegetable kingdom, there is rapid development and speedy decay.

The products of this decomposition readily accumulate, and if not excreted produce their well known effects upon the glands, blood, and nervous centres.

The dietetic and therapeutic principal is obvious.

---

*A Practical Guide to the Study of the Diseases of the Eye: their Medical and Surgical Treatment.* By HENRY W. WILLIAMS, M. D., etc,, etc. Boston, Ticknor & Fields. 1862.

A careful perusal of this unpretending little volume, more than sustains the anticipations with which we announced its reception last month. All the more common and important diseases of the eye, including indeed about all those which the general practitioner will see more than once or twice in a lifetime, are herein clearly, simply and yet graphically described. It gives exactly the information which the active practitioner desires, without any attempt at rhetorical flourish or affectation of extraordinarily profound learning.

We are especially gratified to observe that he treats the eye, as it is, as a part of the whole body with its vitality and functional perfection inextricably interwoven with the rest, and not as a mere mechanical appendage to be fumbled over, cauterized, scarified or lotioned (Egyptian fashion) by itself. He recognizes most thoroughly the simple physiological proposition that the perfection of this delicate organ, as of those more gross, depends upon its nutrition—*the perennial bath of healthy blood.*

We commend the chapter on *Iritis* as illustrating progress —as showing that the writer worships neither formularies gray with age, nor dogmas glittering with mediæval rhetoric.

We remember when mercury was considered the *sine qua non* in ritis—you could *see* its beautiful action as a remedy! All this has gone by—it was the Belladonna that did the work. Just as Calomel has always got the credit due the Opium given with it.

Briefly—we commend this book to our readers' libraries, and better still, to their careful perusal.

---

*Dr. Davis' Institute*—Not the Med. Dep. of "Lind University," but the one whose announcement will be found in our advertising pages. The well-earned reputation of Dr. Davis in his specialty will at once commend his infirmary to the notice of all who have patients of this unfortunate class under their charge. The odor of quackery or undue pretension does not hang around the *New York* Institute. Dr. Davis'

methods have been fully set forth in the medical journals and societies, and the success which has attended them has been very gratifying. It is rare that a general practitioner can treat cases of this kind satisfactorily either to himself or patient, and we believe that they can rely with great confidence upon the facilities offered by the well ordered and appointed "home" herein indicated.

---

*Is there a Lower Deep?*—It appears that the gentleman who does the State of Illinois the honor to direct the expenditure of its war fund; commissions its officers in military and civil life; who appoints examining boards which can scarcely be said to belong to either; and who, it is also whispered, assists personally in the consumption of some of its *cereal* products, has been pestering the War Department for the privilege of foisting upon the volunteer regiments sundry infinitesimal doctors. It turns out, from the published correspondence, that His Excellency in this particular was most disastrously snubbed. Confectionery of this attenuated sort doesn't find a ready market in these war times. We congratulate our readers that some sparkles of common sense still appear on the Black Sea at head-quarters.

But His Excellency was not thus to be put down. The sugar-plum gentry, the eclectics, the clap doctors, the uroscopians, and all that ilk, would not quietly submit. His Excellency had descended to the St. Giles, the Five Points of quackery and folly, and the vermin have naturally enough invested his august person. Their titillations and nibblings, their buzzings and bitings, have put him out of his senses, and he has striven to escape by tossing honey to them, done up in small parcels. The District Surgeoncies for examining candidates for the "exempt brigade," came to hand sweet as molasses. Not "a tub to the whale," but giblets to all Lilliput.

Here and there, *rari nantes in gurgite vasto*, in the list of appointees we see a name which would do credit to the profession in almost any position. Here and there, we see a "regular"—per chance a fossil regular, but still retaining a dim memory of the time when he listened to the Æsculapian patriarchs of Philadelphia. But how did the Governor find time amid his arduous duties to dig down for the rest of the motley crew? Professionally, the list in large part looks like a catalogue of the contents of the witches' cauldron in Macbeth.

